# Micronized Progesterone or Dydrogesterone? A Comparative Study on the Effects of Two Forms of Progesterone on Pregnancy Outcomes After Threatened Abortion

**DOI:** 10.5812/ijpr-136320

**Published:** 2023-11-11

**Authors:** Iman Ansari, Ezzatalsadat Hajiseid Javadi, Hamideh Pakniat, Ali Emami, Fatemeh Ranjkesh, Simindokht Molaverdikhani

**Affiliations:** 1 Department of General Surgery, Shahid Beheshti University of Medical Sciences, Tehran, Iran; 2 Clinical Research Development Unit, Department of Obstetrics and Gynecology, Kowsar Hospital, Qazvin University of Medical Sciences, Qazvin, Iran; 3 Medical Students Research Committee, Qazvin University of Medical Sciences, Qazvin, Iran; 4 Department of Midwifery, Children Growth Research Center, Research Institute for Prevention of Non-Communicable Diseases, Qazvin University of Medical Sciences, Qazvin, Iran; 5 Qazvin University of Medical Sciences, Qazvin, Iran

**Keywords:** Threatened Abortion, Pregnancy Outcome, Progesterone, Dydrogesterone

## Abstract

**Background:**

A significant number of pregnancies are at risk of threatened abortion (TA). Different types of progesterone are used to treat TA.

**Objectives:**

In this study, the effects of 2 forms of progesterone on the continuation of pregnancy and TA-caused pregnancy outcomes were compared.

**Methods:**

A total of 190 women with a gestational age of 6 - 13 weeks presenting with uterine bleeding, closed cervix, and absence of fetal heart rate diagnosed by vaginal examination and ultrasound were allocated into 2 groups and treated with either (D) dydrogesterone (10 mg twice a day) or (M) micronized progesterone (200 mg, twice a day) for beyond 2 weeks after the cessation of uterine bleeding to ensure that bleeding would not recur. The participants were followed up and received prenatal care until the end of pregnancy. The outcomes of pregnancy were recorded and compared between the 2 groups.

**Results:**

The incidence of preeclampsia, gestational diabetes, cesarean section, intrauterine fetal death (IUFD), placenta previa, and abortion was not significantly different between the 2 groups. However, the prevalence of preterm labor and low birth weight (LBW) was significantly lower in M-treated women (P < 0.001 and P = 0.007, respectively). The baby’s weight and gestational age at delivery were significantly higher in the M group than in the D group (P < 0.001). No serious drug side effects were observed in the 2 groups throughout the study.

**Conclusions:**

The results of this study showed that the incidence of preterm labor and LBW was significantly lower in the patients treated with micronized progesterone than in patients treated with dydrogesterone; however, the prevalence of preeclampsia, gestational diabetes, cesarean section, IUFD, and abortion was not significantly different between the 2 groups.

## 1. Background

In the first trimester of pregnancy, any bloody vaginal discharge or uterine bleeding while the cervix is closed is called threatened abortion (TA) (1). Chromosomal abnormalities, thrombophilic disorders, hormonal disorders, anatomical problems of the cervix, etc., are among the most important risk factors for abortion (2). Threatened abortion can indicate placental dysfunction, which may manifest itself in the later stages of pregnancy with a number of complications, such as preeclampsia, fetal growth restriction, low birth weight, preterm labor, and placenta abruption. Threatened abortion occurs in one-fifth of pregnancies, and in half of the cases, it leads to complete abortion (3). The most common treatment for this type of abortion is bed rest and hydration, along with painkillers to help relieve the pain (2, 3). Tocolytic agents, human placental gonadotropin, and a wide range of oral, vaginal, and intramuscular progesterone are used to prevent TA, but the efficacy of these drugs is restricted in many cases (4, 5).

Progesterone is a hormone with undeniable effects on ovulation, implantation, and luteal phase support, playing an important role in pregnancy progression. This hormone facilitates implantation in early pregnancy, regulates the mother’s immune responses, and reduces uterine contractions so the fetus is not rejected (1, 6). In pregnancies in which the corpus luteum is not able to secrete enough progesterone or when the transfer of progesterone to the uterus is disrupted, spontaneous abortion occurs before week 10th of pregnancy (7).

Dydrogesterone is a synthetic derivative of progesterone that is used to treat a variety of problems caused by progesterone insufficiency. Dydrogesterone is pharmacologically very similar to natural progesterone but has good oral tolerance, no adverse effect on normal endometrial secretions, and no inhibiting effects on placental progesterone formation. The micronization of natural progesterone increases its half-life partly due to the production of metabolites such as allopregnanolone, which stimulates progesterone receptors. Also, micronization reduces the particle size of progesterone and enhances its solubility. The uptake of micronized progesterone is doubled when the hormone is consumed with food (8-10).

## 2. Objectives

Different forms of progesterone are produced by pharmaceutical companies around the world. In midwifery, depending on the patient’s wish the physician’s discretion and a specific form of this hormone may be administered. In this regard, what matters is whether these forms can promote different effects independent of the way they are consumed. There is little information about the superiority, side effects, advantages, and disadvantages of various pharmacological forms of progesterone, including for the treatment of TA. Therefore, the aim of the present study was to compare the efficacy of 2 widely used forms of this hormone, dydrogesterone and micronized progesterone, in the treatment of TA.

## 3. Methods

### 3.1. Subjects

This single-blinded randomized clinical trial was conducted on pregnant women referred to Kowsar Women’s Hospital, affiliated with Qazvin University of Medical Sciences (QUMS), Qazvin, Iran, with the diagnosis of TA in a one-year period from October 2019.

Mothers with a gestational age of 6 - 13 weeks presenting with uterine bleeding with a closed cervix on vaginal examination were enrolled. Ultrasound was performed for all patients before participating in the study. Women with fetal or uterine abnormalities, absence of fetal heart rate, multiple pregnancies, hydatidiform mole, pelvic inflammatory disease, and underlying diseases such as cardio-pulmonary disease, thyroid problems, renal or hepatic disorders, and diabetes, as well as those with a history of receiving drug therapy for TA and also mothers who did not consent to participate, were excluded.

**Figure 1.**
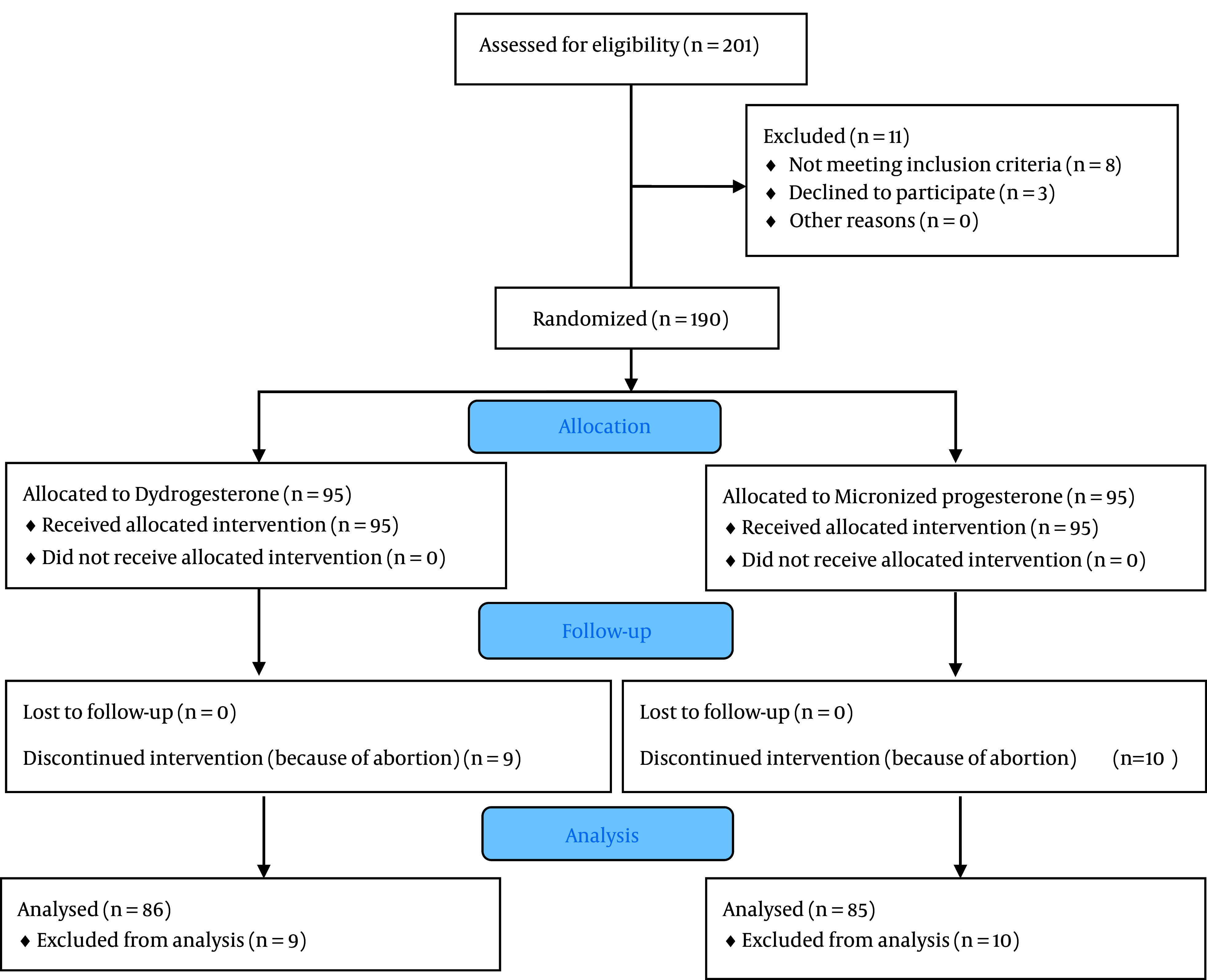
CONSORT flowchart

Participants were randomly assigned to one of the 2 study groups using a random number table to receive the group-based intervention (Figure 1). In group D, 10 mg dydrogesterone tablets (Duphaston® made by Aburaihan Pharmaceutical Co.) were administered twice a day (every 12 hours), and in group M, 200 mg of a micronized progesterone soft gel (Lutogel® made by Tasnim Pharmaceutical Co.) were given twice a day (every 12 hours). The duration of using hormone formulations was from the time of admission to 2 weeks after the cessation of bleeding.

### 3.2. Study Outcomes

Participants were followed up and received prenatal care until the end of pregnancy according to national guidelines. During pregnancy, sequela were recorded and finally compared between the 2 groups. The data collection tool was a checklist, including demographic characteristics such as age, height, weight, body mass index (BMI), gestational age, gravidity, the baby’s weight, and complications, including preeclampsia, preterm labor, low birth weight, gestational diabetes, intrauterine fetal death (IUFD), and abortion. 

### 3.3. Ethical Considerations

The study’s protocol was registered at the Iranian Registry for Clinical Trials (IRCT20120104008611N10), and permission to conduct the study was obtained from the Ethics Committee of QUMS (reference number: IR.QUMS.REC.1398.183). Written informed consent was obtained from all participants after explaining to them the objectives of the investigation. Mothers were assured about the confidentiality of their information and that the study’s outcomes would be published anonymously.

### 3.4. Statistical Analysis

SPSS software version 23 was used to conduct statistical analysis (IBM, Chicago, IL, USA). Quantitative variables were presented as mean ± standard deviation (SD), and qualitative variables were reported as frequency and frequency percentage. The Kolmogorov-Smirnov test was used to check the normal distribution of the data. Pregnancy outcomes were compared between the 2 groups using the independent *t*-test, chi-square test, and Fisher’s exact test at P < 0.05 as the statistical significance level.

## 4. Results

A total of 190 mothers were enrolled in the study and randomly allocated to receive either dydrogesterone (n = 95) or micronized progesterone (n = 95). The mean age of the study’s population was 27.56 ± 6.26 years, and the means of gestational age at the beginning of the study and at the time of delivery were 8.88 ± 1.9 and 34.69 ± 8.35 weeks, respectively. The mean of mothers’ BMIs in this study was 26.47 ± 3.98 kg/m^2^. Table 1 displays the basic characteristics of mothers in the 2 study groups. Overall, 10 mothers (10.5%) in the M group and 9 mothers (9.5%) in the D group experienced a complete abortion, so their obstetric outcomes were not analyzed.

**Table 1.** Basic Characteristics of Mothers in the 2 Study Groups ^a^

Variables	Micronized Progesterone	Dydrogesterone	P-Value
**Mother’s age (y)**	27 ± 6.51	28.13 ± 5.97	0.212
**Mother’s weight (kg)**	67.73 ± 8.26	66.14 ± 12.17	0.11
**Mother’s height (cm)**	164.6 ± 5.33	163.68 ± 5.29	0.24
**Mother’s BMI (kg/m** ^**2**^ **)**	26.96 ± 2.6	27.99 ± 4.53	0.31
**Gestational age at admission (w)**	8.16 ± 1.64	8.61 ± 2.1	0.13
**Nulliparity**	49 (51.6)	42 (44.2)	0.384 ^b^

^a^ Values are expressed as mean ± SD or No. (%).

^b^ Fisher’s exact test.

As shown in Table 2, the incidence of preeclampsia, gestational diabetes, cesarean section, IUFD, and abortion was not significantly different between the 2 groups. Placenta previa was not observed in either group. However, the prevalence of preterm labor and LBW was significantly lower in the M group (P < 0.001 and P = 0.007, respectively), and the means of the baby’s weight and gestational age at delivery were significantly higher in the M group than in the D group (P < 0.001). Also, throughout the study, no serious drug side effects were observed in the 2 groups.

**Table 2.** Comparison of Pregnancy Outcomes Between the 2 Study Groups ^a^

Variables	Micronized Progesterone	Dydrogesterone	P-Value
**Gestational age at delivery (w)**	38.77 ± 1.79	35.68 ± 4.07	< 0.001 ^b^
**Baby’s Weight (g)**	3324.58 ± 546.15	2734.76 ± 784.42	< 0.001 ^b^
**Cesarean section**	25 (29.4)	33 (38.3)	0.27 ^c^
**Gestational diabetes**	7 (8.2)	7 (7.4)	1 ^c^
**Preeclampsia**	9 (10.6)	11 (11.6)	0.814 ^c^
**Intrauterine fetal death**	0 (0)	3 (3.2)	0.246 ^c^
**Placenta previa**	0 (0)	0 (0)	-
**Preterm birth (before week 36th)**	10 (11.8)	38 (44.2)	< 0.001 ^c^
**Abortion**	10 (10.5)	9 (9.5)	1 ^c^
**Low birth weight**	13 (15.3)	29 (33.7)	0.007 ^c^

^a^ Values are expressed as mean ± SD or No. (%).

^b^ Independent *t*-test.

^c^ Fisher’s exact test.

## 5. Discussion

Ovulation, implantation, and luteal phase support, which are required for the continuation of pregnancy, are orchestrated by progesterone. In addition to its role in supporting the luteal phase and assisted reproductive technology, progesterone also plays a role in the treatment of TA, as well as in preventing recurrent miscarriage and preterm labor (11, 12). By facilitating implantation in early pregnancy, progesterone regulates the mother’s immune responses and reduces uterine contractions, helping maintain the fetus (1). Although the primary source of progesterone for the continuation of pregnancy is the ovary, this hormone starts to be secreted by the placenta, and its level gradually increases during pregnancy. The results of studies indicate a decrease in serum progesterone levels in patients with TA compared to women experiencing normal pregnancies, suggesting progesterone insufficiency as an independent risk factor of abortion (13, 14). Therefore, due to the importance of preventing TA and also the possible role of progesterone in improving placental vascular function and preventing abortion, we decided to compare the effects of oral micronized progesterone and oral dydrogesterone on pregnancy outcomes in women presenting with TA.

The results of the present study demonstrated that the incidence of preterm labor and LBW was significantly lower in pregnant women treated with micronized progesterone, while the baby’s weight and gestational age at delivery were significantly higher in them compared to the mothers treated with dydrogesterone. On the other hand, the prevalence of preeclampsia, gestational diabetes, cesarean section, IUFD, and abortion was not significantly different between the 2 groups.

In comparison, Pandian (15) in 2009 showed that the incidence of abortion was significantly lower in patients treated with dydrogesterone (12.5%) than in the counterparts receiving standard treatment (28.4%). Also, pregnancy outcomes, including cesarean section, preterm labor, placenta previa, preeclampsia, prenatal bleeding, and LBW, were comparable between the 2 groups. Consistent with the results of the recent study, regardless of the fact that the comparison group received a different treatment in Pandian’s study (15), we recorded only 9 (9.5%) cases of abortion, which this relatively low rate could be due to the beneficial effects of dydrogesterone in maintaining pregnancy. In addition, in 2014, Kumar et al. (16) showed that dydrogesterone treatment could improve pregnancy outcomes, such as gestational age at delivery, the infant’s weight, and the incidence of abortion. Also, the results of a systematic review conducted by Carp (17) in 2012 showed that the frequency of abortion in pregnant women treated with dydrogesterone was 13% compared to 24% in the placebo group, supporting the beneficial role of this medication in preventing abortion, as observed in the present study.

In addition, we also observed almost the same results in women treated with micronized progesterone. In a study by Turgal et al. (18) in 2016, it was found that hormonal support with micronized progesterone in patients with TA significantly increased placental volume and thus considerably contributed to the maintenance of pregnancy and reduced rate of abortion. Also, the results of a meta-analysis study by Coomarasamy et al. (19) in 2020 showed that micronized progesterone significantly increased pregnancy maintenance in women with TA compared to the placebo. Another meta-analysis study by Devall et al. (20) in 2021 revealed that in mothers with a history of recurrent miscarriage, micronized progesterone could significantly increase the rate of live births. Overall, the results of these studies are consistent with our findings in the present study.

Another point of focus in this study was to compare the effectiveness of these 2 forms of progesterone in the treatment of TA. In this regard, Siew et al. (21) (2018) compared the therapeutic efficacy of micronized progesterone and dydrogesterone and reported that the incidence rate of miscarriage was 10.1% in the micronized progesterone group and 15.2% in the dydrogesterone group, but this difference was not statistically significant. This finding was similar to the results of the present study. Although in the present study, the incidence of abortion was not significantly different between the 2 groups, the incidence of preterm labor and LBW in the micronized progesterone group was significantly lower compared to the dydrogesterone group. Czajkowski et al. (22) (2007) compared uteroplacental circulation between the patients treated with either micronized progesterone or dydrogesterone, reporting lower spiral artery pulsatility, resistance index, and systolic/diastolic ratio in the former group, while dydrogesterone treatment was only associated with a decrease in the uterine artery systolic/diastolic ratio.

A clinical trial by Pakniat et al., who assessed the effect of vaginal progesterone and dydrogesterone on pregnancy outcomes in patients with TA, showed that dydrogesterone and vaginal progesterone had comparable impact on the occurrence of pregnancy outcomes and maternal and neonatal complications. Considering the similar efficacy of these drugs, either of them can be chosen based on factors such as the patient’s allergies, accessibility of the drug, and affordability (9).

Therefore, it seems that the better effects of micronized progesterone in this study are probably due to the improvement of uteroplacental circulation, which ultimately leads to better pregnancy maintenance and prevents preterm labor and LBW in mothers with TA. According to the results of the present study and other similar studies, it seems that both forms of progesterone are effective in treating TA and reducing abortion and other TA-related sequela during pregnancy. However, micronized progesterone is probably more effective in reducing preterm labor and LBW, probably due to better improvement in uteroplacental circulation. Among other factors, the cost of the drug, the mother’s preference, and possible treatment side effects can influence the choice of the drug form.

### 5.1. Conclusions

The results of this study showed that the incidence of preterm labor and LBW was significantly lower in the pregnant mothers treated with micronized progesterone than in their counterparts treated with dydrogesterone. Nevertheless, the prevalence of preeclampsia, gestational diabetes, cesarean section, IUFD, and abortion was not significantly different between the 2 groups.

## Clinical Trial Registration Code:

IRCT20120104008611N10.
